# Optimization and characterization of polyhydroxybutyrate produced by *Halomonas meridiana* using orange peel waste

**DOI:** 10.1186/s12866-025-04007-2

**Published:** 2025-05-19

**Authors:** Mahmoud H. Hendy, Amr M. Shehabeldine, Amr H. Hashem, Ahmed F. El-Sayed, Hussein H. El-Sheikh

**Affiliations:** 1 Botany and Microbiology Department, Faculty of Science, Al-Azhar University, Cairo, 11884 Egypt; 2 Microbial Genetics Department, Biotechnology Research Institute, National Research Centre, Giza, Egypt; 3 Egypt Center for Research and Regenerative Medicine (ECRRM), Cairo, Egypt

**Keywords:** Polyhydroxybutyrate, Response surface optimization, *Halomonas meridiana*, Halophilic bacteria

## Abstract

The production of bioplastics from marine microorganisms is significantly relevant in the field of biotechnological applications for sustainable ecological management. Nevertheless, the expense associated with PHB production is substantial and regarded as the primary obstacle to its industrialization. In this study, orange peel waste served as a carbon source to enhance PHB production efficiency. Among the 15 strains evaluated, MH 96 was selected for PHB production due to its high salt tolerance and efficient utilization of orange peel as a substrate. The highest producing PHB strain MH96 was genetically identified using 16S rRNA sequencing as *Halomonas meridiana* and submitted in the GenBank under accession numbers PP826284. The optimal fermentation conditions were evaluated through single-factor optimization. Upon completion of the response surface optimization, the Plackett–Burman and Box-Behnken design experiments were conducted utilizing the outcomes of the single-factor optimization. The final parameters were the inoculum size of 1.74, (NH_4_)_2_HPO_4_ concentration of 1.0 and pH 6.37, and PHB yield of 5.94 g/L. The characterization of the extracted biopolymer by NMR, FTIR, XRD, and thermal properties was used to examine the properties of the extracted PHB, and gas chromatography-mass spectrometry (GC–MS) proves the presence of 2-butenoic acid, 1-methyl ethyl ester, tetradecane, hexadecanoic acid, methyl ester, and docosanoic acid, 8,9,13-trihydroxy-. Methyl ester, which confirmed the structure of the polymer as PHB.

## Introduction

The widespread production of synthetic plastics, coupled with existing legislative frameworks, has intensified the quest for bio-based and biodegradable polymers. Among these, polyhydroxyalkanoates (PHAs) are notable biodegradable polyesters synthesized intracellular by various microorganisms [1]. These biopolymers typically manifest as storage granules within microbial species, accumulating when there is an imbalance in nutrient levels, thereby serving as energy reserves and a survival strategy for the microorganisms.

PHAs can be categorized based on the number of carbon atoms in their hydroxyalkanoate monomers: short-chain length (scl-PHAs, C_1_-C_5_), medium-chain length (mcl-PHAs, C_6_-C_14_), and long-chain length (lcl-PHAs, > C_14_). The physicochemical properties of these polymers are influenced by their composition; scl-PHAs exhibit properties akin to polyethylene (PE) and polypropylene (PP), while mcl-PHAs share characteristics with rubber and elastomers [1, 2].

PHAs are a better alternative to bioplastic than petroleum-based polymers [3]. The biocompatibility and biodegradability of PHAs bioplastics enable a range of uses in both agriculture and medicine [4]. Poly (3-hydroxybutyrate) (PHB) is a biopolymer synthesized through microbial cultivation, requiring efficient microorganisms and cost-effective substrates. The moderately halophilic bacterium *Halomonas boliviensis* LC1 has demonstrated high PHB production efficiency, utilizing leftover quinoa (*Chenopodium quinoa* Willd) as a substrate [5].

Halophiles possess the remarkable ability to thrive in high-salinity environments where most other organisms would not survive. Among these halophilic species, certain actinomycetes, cyanobacteria, and yeast are known to produce polyhydroxybutyrate (PHB), a biopolymer that can serve as a substitute for synthetic polymers in various commercial applications [6].

Previous study has examined the presence of PHB derivatives in ten strains of the genus *Streptomyces* within the actinomycetes group. Nevertheless, there remains a limited body of literature focused on using actinobacteria to synthesize PHB [7]. Numerous investigations have looked into the possibility of genetically modifying halotolerant bacterial strains to increase their capacity to produce PHA [8], and using them in industrial biotechnology [9].

These results in the cost-effectiveness of the substrates employed as the carbon source in PHA production. Thus, the most crucial strategy for reducing production costs is to use cheap raw materials [10]. For this reason, to make biopolymer-producing microorganisms financially viable, it is preferred to culture them using inexpensive raw resources, such as agricultural and agro-industrial residues [11].

Orange peel waste represents a valuable nutrient source for bacteria that produce PHB [12], due to its composition, which includes soluble and insoluble carbohydrates and a relatively low protein content [13]. This results in a favorable carbon-to-nitrogen ratio. Additionally, with an estimated global production of 10 million tons annually, orange peel waste constitutes the primary byproduct of the citrus processing industry [14, 15]. Furthermore, PHA production can be improved by optimizing procedure factors utilizing a variety of tools for optimization [16].

Previous research has utilized the Response Surface Methodology (RSM) to enhance the production rate of PHAs by various bacteria, including *Rhodobacter sphaeroides*, *Bacillus coagulans,* and *Ralstonia eutropha*. Utilization is readily available; the current increase in Gram-positive bacteria stains has been promoted by low-cost substrates. in optimizing bacterial requirements for optimal PHA production [17]. By screening and examining the interactions between the parameters, a factorial design employing RSM is utilized to show the cumulative effect of the factors. Regarding low-cost carbon sources, statistical optimization approaches remain a crucial tactic for achieving an ideal PHA concentration immediately before large-scale manufacturing [18].

The aim of this study is to enhance the production efficiency of polyhydroxybutyrate (PHB), a bioplastic, from marine microorganisms using orange peel waste as a sustainable carbon source. The study focuses on isolating and identifying high PHB-producing strains, optimizing fermentation conditions through single-factor and response surface methodologies, and characterizing the extracted biopolymer to confirm its structural and thermal properties. The ultimate goal is to reduce the production costs associated with PHB and overcome the barriers to its industrialization, thereby contributing to sustainable ecological management through biotechnological applications.

## Materials and methods

### Isolation sources and medium

Water and soil samples were collected from the Max salt pans in Alexandria, Egypt, using a TYS medium with the following composition (g/L): NaCl 75, KCl 0.7, CaCl_2_·2H_2_O 1.4, MgSO_4_·7H_2_O 6.8, MgCl_2_·6H_2_O 5.4, NaHCO_3_ 0.2, yeast extract 0.5, and peptone 1. Sucrose was added as the sole carbon source at 20 g/L, and the pH was adjusted to 7.0 using 1 N HCl and 1 N NaOH [19].

### Screening tests for PHB -producing isolates

To screen for PHB -producing bacteria, a solid medium was supplemented with 0.5 µg/mL of Nile Red. A sterilized viable colony staining technique was applied to Petri dishes. After three days of incubation at 37 °C, the isolates were streaked onto agar plates and examined under UV light, with positive colonies identified by fluorescence [20]. The cultures were maintained at a pH of 7.0 on nutrient agar plates at 37 °C for one day to preserve PHB -producing bacteria. A confirmation test for PHB production was conducted using Sudan Black-B (SBB) stain [21]. A subset of isolates was cultivated in TYS medium supplemented with 2% sucrose, incubated at 37 °C and 150 rpm for 72 h, after which polymer measurements were taken following extraction [22].

### Identification of the most potent bacterial isolation

#### Biochemical and morphological characteristics

To identify the most potent isolation MH 96, the morphological (shape, Gram reaction) were examined.

#### 16S rRNA sequencing and phylogenetic analysis

Genomic DNA was isolated from the overnight culture of chosen isolates using a Qiagen DNA purification kit, according to the manufacturer's guidelines (Qiagen, Hilden, Germany). PCR amplification of 16S rRNA was conducted using genomic DNA from the isolates as a template. Two primers were utilized: 8 F (5'-AGAGTTTGATCCTGGCTCAG-3') and 1495 R (5'-CTACGGCTACCTTGTTACGA-3'), along with GoTaq Flexi DNA Polymerase (Promega, WI, USA), following the protocol established by [23]. The resulting sequences were edited and shortened using BioEdit version 7.2.5. Subsequently, these modified sequences were aligned with those in the GenBank database through BLAST analysis. A phylogenetic tree was constructed based on the 16S rRNA sequences using the UPGMA method. The percentage of replicate trees in which the associated taxa clustered together in the bootstrap test (1000 replicates). The tree is drawn to scale, with branch lengths in the same units as those of the evolutionary distances used to infer the phylogenetic tree. The evolutionary distances were computed using the Maximum Composite Likelihood method [24, 25].

### Factors affecting PHB production (single factor optimization).

This experiment examined the effects of salinity, nitrogen supply concentration, orange peel concentration, and peptone and yeast extract concentration to improve the culture medium. Table 1 enumerates the particulars of the various sets. After fermentation, the bacteria were examined to determine the PHB yield, and the DCW and PHB After yields were utilized to determine the optimal single-factor conditions. The investigation also examined the impacts of temperature, starting pH, agitation speed, incubation duration, and inoculum size to optimize fermentation conditions [26, 27].

**Table 1:** Gradient setting of each factor in single factor optimization experiment

Concentration gradient setting											
**Media single-factor optimization**	Orange peel (g/L)	100	200	300	400	500	600	700	800	900	1000
	Type of nitrogen source	NH_4_Cl	1.5	-	-	-	-	-	-	-	-
		(NH_4_)_2_SO_4_	1.5	-	-	-	-	-	-	-	-
		(NH_4_)_2_HPO_4_	1.5	-	-	-	-	-	-	-	-
		NaNO_3_	1.5	-	-	-	-	-	-	-	-
		Casein	1.5	-	-	-	-	-	-	-	-
		Peptone	1.5	-	-	-	-	-	-	-	-
		Yeast extract	1.5	-	-	-	-	-	-	-	-
		yeast extract &peptone	1.5	-	-	-	-	-	-	-	-
**Fermentation condition single-factor optimization**	(NH_4_)_2_HPO_4_ (g/L)		0.5	1	1.5	2	2.5	3	4	-	-
	Incubation time (h)		12	24	36	48	60	72	84	96	-
	Inoculation size (%) (v/v)		1	2	4	6	8	10	12	-	-
	Initial pH		5	6	6.5	7	7.5	8	9	10	-
	Salinity (%)		0	2	5	7.5	12.5	15	17.5	20	-
	Temperature (^o^C)		25	30	35	37	40	45	50	-	-
	Agitation rate (rpm)		0	50	150	200	250	-	-	-	-

#### Optimization of PHB using Plakett–Burman (PB) design

To identify significant factors influencing PHB production, a Plackett–Burman (PB) experimental design was employed [28, 29]. As shown in Table 2, eleven variables related to medium composition and culture conditions were examined at low (− 1) and high (+ 1) levels. This two-level factorial design allows for the investigation of 12 variables (n + 1) [30], and to minimize errors, three replicated center points were included. The anticipated response (Y) can be modeled as:

1 $$Y=\beta 0+\sum \beta ixi$$

**Table 2:** The levels of each factor in the plackett–burman experimental design

Factor code	Factor	Level value	
		**Low (− 1)**	**High (+ 1)**
**A**	Orange peel concentration (g/L)	600	1000
**B**	Temperature (^o^C)	35	40
**C**	Sodium chloride (%)	10	15
**D**	Incubation time (hr.)	72	96
**E**	Inoculum size (%) (v/v)	2	10
**F**	Agitation rate (rpm)	120	180
**G**	MgCl_2_.6H_2_O (g/L)	1.35	4.05
**H**	MgSO_4_.7H_2_O (g/L)	1.7	5.1
**J**	(NH_4_)_2_HPO_4_ (g/L)	1.0	3.0
**K**	Methanol (co-substrate) (g/L)	2.0	4.0
**L**	Initial pH	5	8

Y is the anticipated response, β_0_ is the model intercept, β_i_ denotes the linear coefficient, and X_i_ indicates the levels of the independent variables. PHB production was measured in triplicate, and the response was calculated as the average of these values. Variables with significant effects at the 95% confidence level (*p* < 0.05) were identified for further optimization.

#### Response surface methodology

To assess process stability and variability, a second-order response surface was created using a Box-Behnken design (BBD), which included three factors, three levels, and three replicates at the center point [31]. The selection of center points followed the Plackett–Burman design framework. Table 3 outlines the three levels: high (+ 1), middle (0), and low (− 1). The data obtained were analyzed using regression analysis with the"Design Expert"software (Version 7.0). The accuracy of the polynomial model was evaluated using the coefficient of determination (R^2^). Each experimental trial was conducted in triplicate. The second-order polynomial regression model can be represented as follows:

2 $$Y={\beta }_{0}+\sum {\beta }_{ixi}+\sum {\beta }_{iixi}^{2}+{\beta }_{ijxixj}+\Sigma$$

**Table 3:** The levels of each factor in the box-behnken experimental design

Factor	Level value		
	** − 1**	**0**	** + 1**
Inoculum size (%) (v/v)	2	4	6
(NH_4_)_2_HPO_4_ (g/L)	1.0	1.5	2
Initial pH	6.5	7	7.5

In this equation x_i_ and x_j_ are the coded independent variables, Y is the expected response, β_0_ is the intercept, β_i_ is the linear coefficient, β_ij_ represents the interactive coefficients, β_ii_ denotes the quadratic coefficients, and ∑ accounts for the error term.

### Characterization of PHB synthesis by the most potent bacterial isolate

#### Gas chromatography-mass spectrometry (GC–MS) detection

Research at the National Research Center in Dokki, Egypt, employed a direct capillary column (30 m long, 0.25 µm thick, 25 mm internal diameter) with a Trace GC1310-ISQ mass spectrometer [32]. For analysis, air-dried biomass or pure PHB was placed in a glass tube with 1 ml of chloroform, 850 μl of methanol, and 150 μl of H_2_SO_4_. The tube was sealed and hydrolyzed at 100 °C for 160 min. After hydrolysis, an equal volume of water was added, and the mixture was stirred. A 2 μl sample was taken from the bottom layer for injection, using benzoic acid as an internal standard. In this study, PHB identification was confirmed by analyzing GC–MS results and comparing them with previously published data that used standard PHB as a reference for spectral analysis. The similarity in characteristic peaks between our samples and the standard PHB validates the polymer's identity [33].

#### Fourier transform infrared chromatography analysis (FTIR)

FTIR spectroscopy was used to analyze the functional groups in the isolated PHB at the National Research Center. The biopolymer was dissolved in chloroform and mixed with potassium bromide (KBr) pellets, with the solvent removed afterward. Infrared spectra were recorded using an FTIR/4 Jascoo across a wave number range of 400 to 4000 cm⁻.^1^ [34]. In this study, PHB identification was confirmed by analyzing FTIR results and comparing them with previously published data. The methodology followed that of [35], where standard PHB was used as a reference for spectral analysis. The similarity in characteristic absorption peaks between our samples and the standard PHB supports the polymer identity [35]

#### Nuclear magnetic resonance analysis (NMR)

Twenty-five milligrams of dried PHB samples were dissolved in 100 μL of deuterochloroform (CDCl₃), and the chemical structure was analyzed using both ^1^H and ^13^C NMR on a 500 MHz spectrometer [34].

#### X-ray diffraction analysis (XRD)

Purified PHB samples were compressed onto a glass slide for XRD analysis. The measurements were taken using a Bruker D8 Advance diffractometer with a copper source at 40 mA and 40 kV, over a 2θ range of 5º−80º with a step size of 0.05º[36]. In this study, PHB identification was confirmed by analyzing XRD results and comparing them with previously published data. Penkhrue et al. (2020), where standard PHB was used as a reference for spectral analysis [35]

#### Thermogravimetry analysis (TGA)

A 5 mg sample was analyzed using the Themsys One Plus SETARAM under a nitrogen atmosphere, with a flow rate of 20 mL/min and a heating rate of 10 °C/min. Thermogravimetric analysis (TGA) and differential thermogravimetry (DTG) were performed to evaluate the polymer's thermal stability and degradation temperature [37].

#### Differential scanning calorimetry) DSC)

DSC analyses were conducted on all polymer samples under nitrogen to determine glass transition (Tg) and melting temperature (Tm). The procedure included three temperature cycles: heating from room temperature to 250 °C, cooling to −50 °C, and reheating back to 250 °C, with specified heating and cooling rates [35].

### Analytical methods

#### Determination of cell dry weight

The optical density at 600 nm was measured using a spectrophotometer (M-ETKAL-721) to evaluate cell growth. Following a five-minute centrifugation at 10,000 rpm and 4 °C, the cell pellet was washed with distilled water. To ensure constant weight, the pellet was dried overnight at 105 °C. A standard calibration curve correlating OD_600_ with cell dry weight was used to ascertain cell mass concentration. [20].

#### Determination amount of PHB

The extraction of PHB from bacteria involved centrifuging the samples at 10,000 rpm for 10 min to collect the bacterial cells after incubation. The resulting pellet was treated with a 4% sodium hypochlorite solution and incubated at 37 °C for one hour, followed by a 15-min centrifugation at 5000 rpm. After washing with distilled water and cleaning with acetone, the pellet was dissolved in 5 ml of boiling chloroform, which was allowed to evaporate [38]. The extracted PHB was then analyzed spectrophotometrically by adding 10 ml of concentrated H₂SO₄ to the chloroform solution, sealing it, and heating it for 10 min. After cooling, the solution was analyzed using a UV spectrophotometer at 235 nm, with a standard curve created for PHB concentrations between 0.5 and 3.5 mg/ml.

### Statistical analysis

All experiments were conducted in triplicate, with means and standard deviations reported. A stepwise regression approach was used to formulate an optimal model, and ANOVA was applied to analyze the main effects of the fitting model, defining significance as *P* < 0.05. Data analysis was performed using Design Expert 7.0 and Excel 365.

## Results and discussion

### Isolation and screening of PHB -producers from different sources

As described in the materials and methods, PHB -producing bacterial isolates were obtained using enrichment media supplemented with glucose. Samples were collected from various liquid and solid sources across different locations in Egypt. A total of 15 bacterial isolates were isolated and analyzed. Qualitative screening for PHB production was conducted using the Nile red staining assay, revealing that one bacterial isolate MH46 exhibited significant accumulation for PHB but other isolates showed weak production.

Another qualitative screening for PHB production using Sudan Black B staining method to assess their PHB production. Dark, blue-colored colonies that produced PHB were regarded as promising candidates for the synthesis of PHB. Results revealed that bacterial isolate MH96 was the most potent for PHB production. To confirm the qualitative results, quantitative screening was carried out on all bacterial isolates to quantify PHBTo extract PHB, the cells were subsequently collected, and evaluated, and the PHB content was compared. Table (4) reveals that bacterial isolate MH 96 produced PHB 0.49 g/L, when using sucrose as a carbon source while this isolated produced PHB with1.0 g/Lin the case of oeange peel as substrate. Cost-effective manufacturing necessitates the availability of inexpensive renewable sources for carbon feedstock and bacterial strains capable of generating substantial quantities of intracellular PHB utilizing these economical substrates. These wastes are abundantly accessible and provide substantial sources of carbohydrates generated by the agricultural industry. The six most potent halo-bacteria isolates selected were cultivated in a TYS medium containing 10% sodium chloride and glucose. Consequently, the isolate MH 96 was employed as the most effective halo-bacterium for production and optimization. These wastes are abundantly accessible and serve as substantial sources of carbohydrates generated by the agricultural sector. Consequently, the isolate MH 96 was employed as the most effective halo-bacterium for production and optimization. These wastes are abundantly accessible and serve as substantial sources of carbohydrates generated by the agricultural sector.

**Table 4:** Quantitative primary screening test using sucrose and orange peel as substrates, 15 halophilic bacterial isolates were selected for PHB production

No	Isolates Code	PHB (g/L) ± SD from:	
		**Sucrose**	**Orange peels**
**1**	MH 47	0.280 ± 0.037	0.783 ± 0.007
**2**	**MH 96**	**0.490 ± 0.020**	**1.000 ± 0.011**
**3**	AD 1–1	0.18505 ± 0.0087	0
**4**	AD 1–3	0.278 ± 0.006	0
**5**	AD 4–1	0.1250 ± 0.0042	0
**6**	AD 4–2	0.18742 ± 0.0059	0
**7**	AD 4–3	0.1580 ± 0.0051	0
**8**	AD 4–4	0.2060 ± 0.0026	0
**9**	AD 4–5	0.0822 ± 0.0035	0
**10**	AD 6–1	0.2605 ± 0.0004	0
**11**	AD 7–3	0.0563 ± 0.0059	0
**12**	AD 8–1	0.1685 ± 0.0042	0
**13**	AD 9–1	0.0855 ± 0.0052	0
**14**	AD 10–1	0.2749 ± 0.0051	0
**15**	AD 10–3	0.1820 ± 0.0040	0

The production of PHB by bacteria from waste has been previously reported. For instance, the newly isolated *Bacillus flexus* strain AZU-A2 was used for the production and optimization of polyhydroxyalkanoates using sugarcane molasses as a cost-effective substrate [20]. Additionally, [39] described the production and optimization of polyhydroxylic acids using glycerol as a substrate with the newly isolated *Zobellella taiwanensis* Azu-IN1 strain at 37 °C and 1% (v/v) glycerol. Furthermore, *Halomonas alkaliantarctica* was reported to produce PHB using dairy waste as a substrate [40].

### Identification of the most potent bacterial isolate MH 96

#### Morphological and biochemical characteristics

**Fig. 1:**
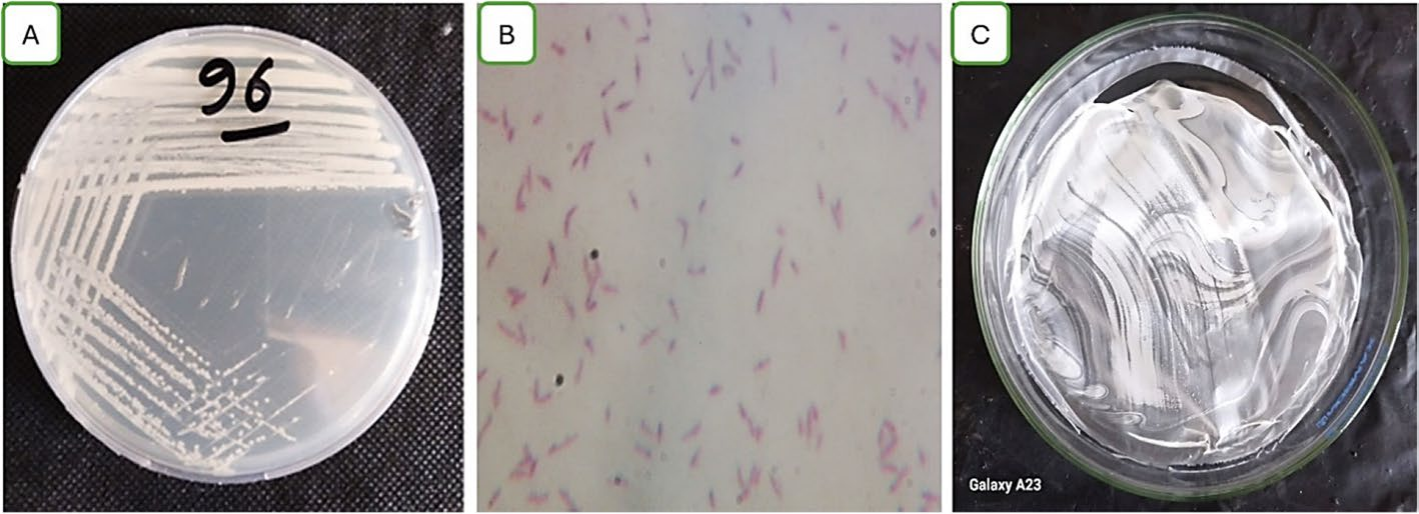
Shape of bacterial isolation MH 46 on an agar plate (A), under the light microscope (B), and (C) shows PHB after extraction from strain MH 46

The leather-producing enterprise generated soil from which MH 96 was extracted. MH 96 was identified by examining the isolates'morphology (Gram reaction, shape), biochemistry, and additional features. The strain MH 46 is rod-shaped, Gram-negative, has positive catalase activity (Fig. 1) and has a negative KOH reaction. It can flourish with up to 10% sodium chloride or elevated salinity.

#### Molecular identification and phylogenetic analysis

**Fig. 2:**
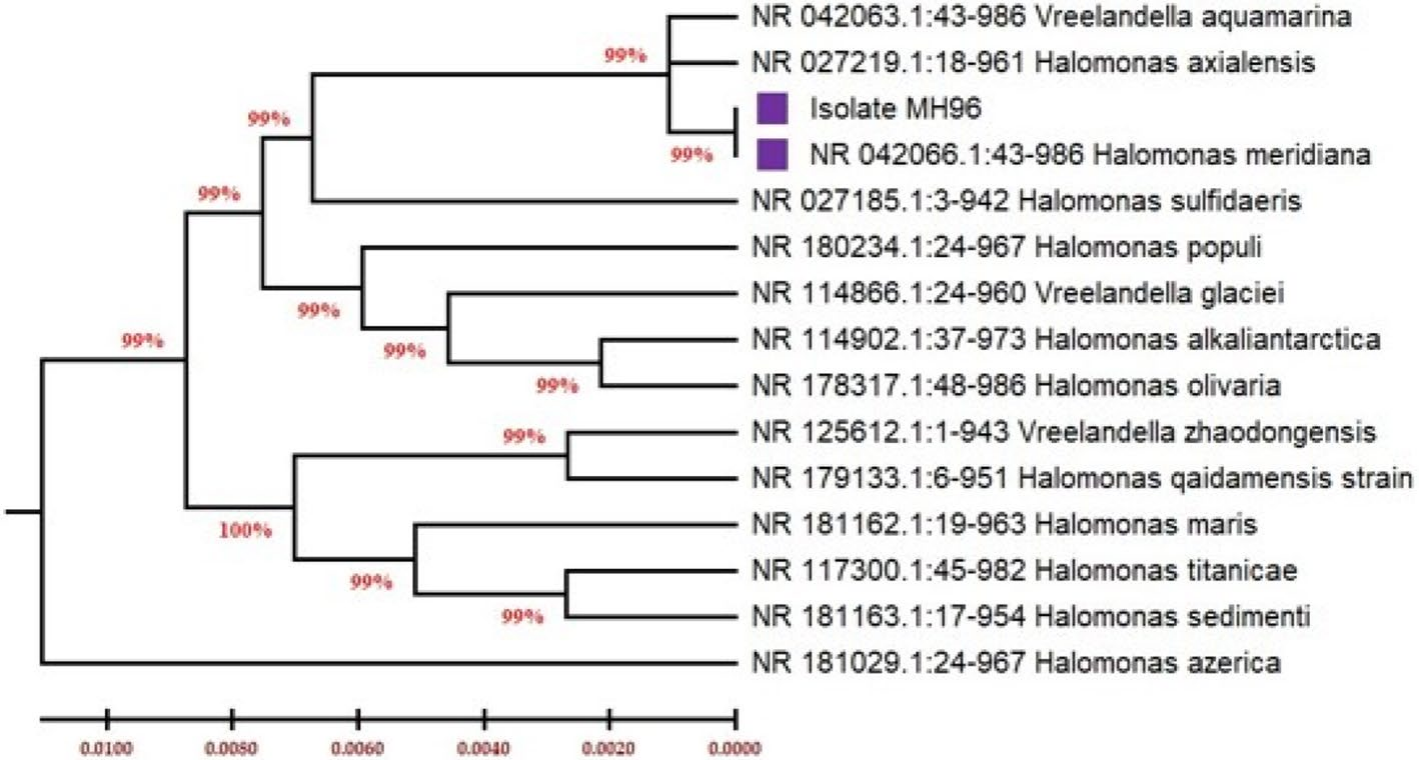
Phylogenetic tree derived from 16S rRNA gene sequences illustrating the relative position of strain for *H. meridiana* MH 46. The tree was constructed using MEGA 6.06

The 16 S rRNA gene of the bacterium MH 96 was amplified and sequenced, enabling molecular identification. Following purification, sequencing, and alignment, blast analysis was used to compare the amplified PCR products to published sequences of 16 S rRNA gene strains kept in NCBI databases. The isolate with more than 99% similarity in its blast findings led to its identification as *Halomonas meridiana*. With accession code PP826284, the capacity of the genus *Halomonas* to synthesize PHB from various carbon sources is recognized [41, 42], (Fig. 2) shows its phylogenetic tree.

### Optimization of PHB production using the one-factor-at-a-time (OFAT) Approach

**Fig. 3:**
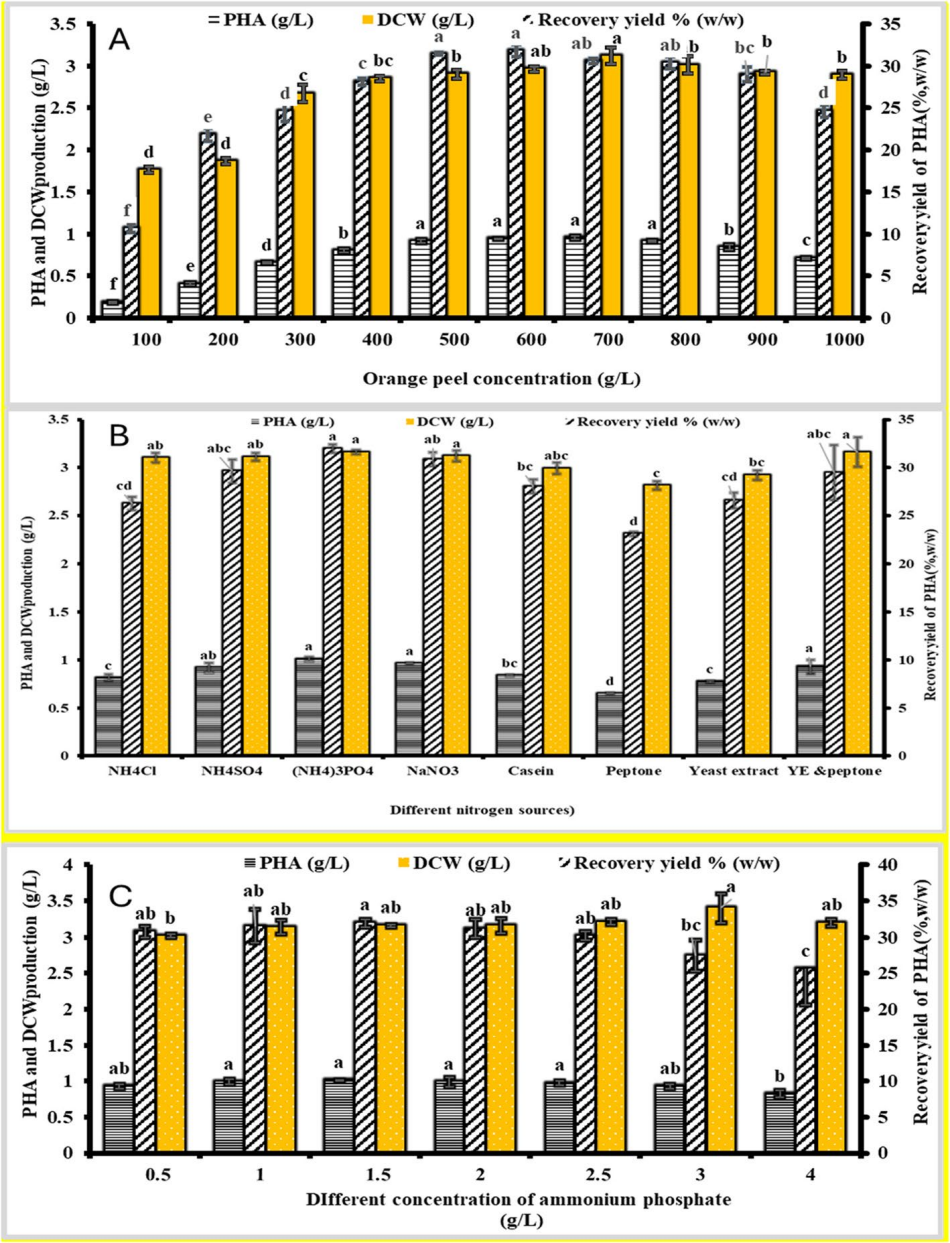
Influence of medium constituents on PHB production by strain MH 96. A. Influence of orange peel concentration on PHB yield. B. Influence of nitrogen source type on PHB yield. C. Influence of peptone and yeast extract concentration on PHB yield. Each value is mean of 3 replicates ± standard error of means. Different lower-case-letters in the same bars are significantly different by post hoc-Tukey’s Honestly Significant Difference test (HSD) at p ≤ 0.05, values of the same bars with the same letters are not significantly different

*Halomonas* sp. can synthesize polyhydroxyalkanoates (PHB) without requiring nitrogen sources, magnesium ions, sulfate ions, or other additional components, as long as a sufficient carbon supply is available [43]&[42]. In this study, single-factor optimization experiments were conducted to evaluate the effects of (NH₄)₂HPO₄ concentration, orange peel concentration, and nitrogen source type on the production of polyhydroxybutyrate (PHB) by strain MH 96 (Fig. 3). As shown in Fig. 3A, strain MH 96 was cultivated in TYS medium at 37 °C and pH 7.0, with initial orange peel concentrations ranging from 100 to 1000 g/L. The dry cell weight (DCW) increased at 100 g/L and 700 g/L, reaching a maximum at 700 g/L, where PHB production peaked at 0.95 g/L with a recovery yield of 30.57%. However, at 1000 g/L, a slight decline in DCW was observed, suggesting that 700 g/L is the optimal concentration for PHB fermentation, though the relatively low overall productivity indicates room for further optimization.

The impact of diverse organic and inorganic nitrogen sources on growth and PHB synthesis was evaluated, as illustrated in Fig. 3B and C. Cell growth increased with fermentation duration, reaching a peak of 3.16 g/L at 96 h with ammonium phosphate. While similar DCW (range from 2.81 to 3.16 g/L) was achieved with alternative nitrogen sources, the maximum recovery yield of 31.96% (w/w) was attained with ammonium phosphate. The maximum PHB achieved was 1.01 g/L, with a recovery yield of 31.96. An ideal concentration of 1.5 g/L was identified for maximizing PHB production efficiency.

**Fig. 4:**
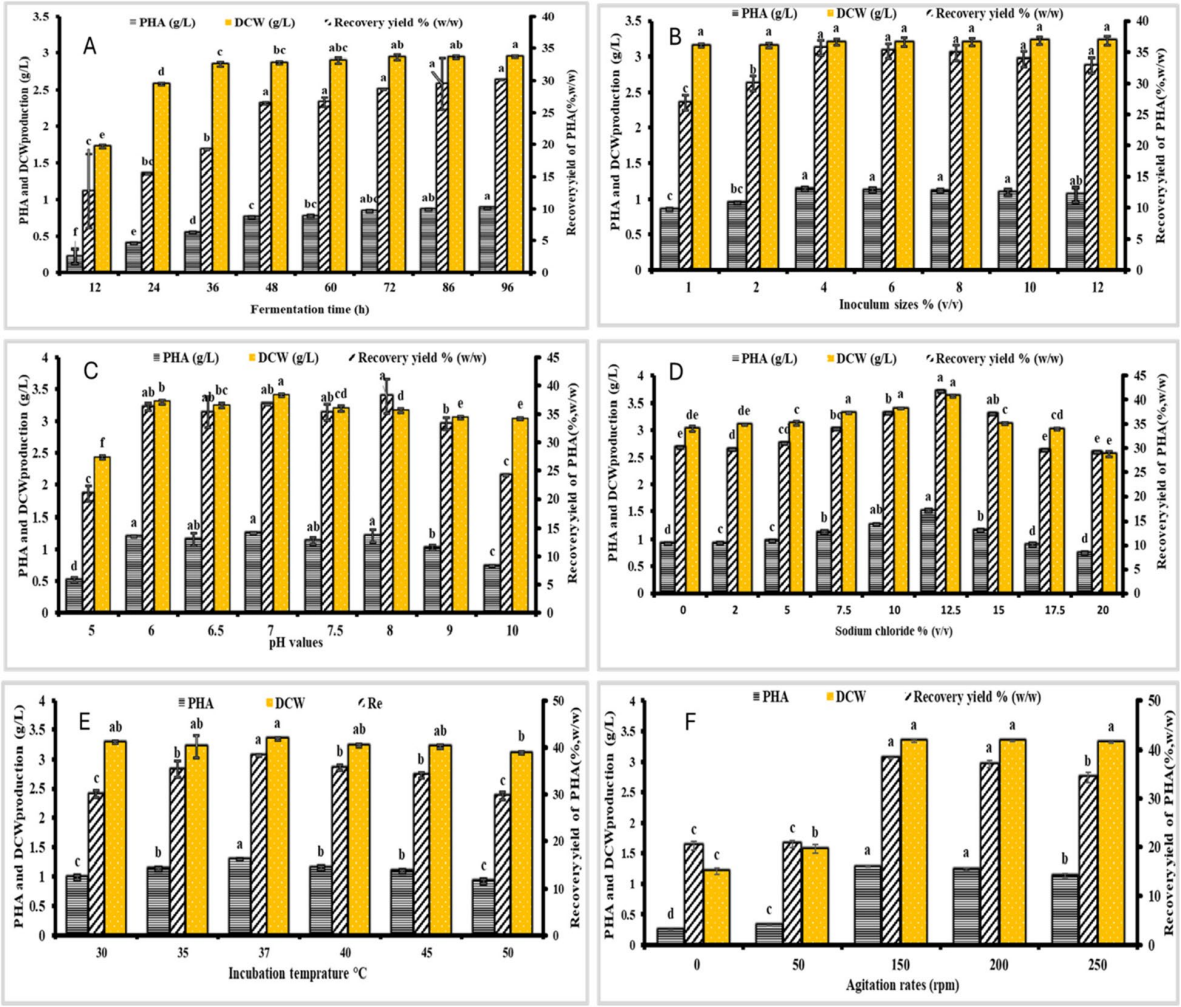
Effect of various factors on PHB production by strain MH 96. (A) Fermentation duration. (B) Inoculum size. (C) pH. (D) Sodium chloride concentration. (E) Temperature. (F) Agitation rate and inoculum size. Each value represents the mean of three replicates ± standard error of the mean. Different lowercase letters within the same bars indicate significant differences according to Tukey’s Honestly Significant Difference (HSD) test (p ≤ 0.05), whereas bars with the same letters are not significantly different

Experiments were undertaken to optimize temperature, agitation rate, pH, sodium chloride concentration, inoculum size, and fermentation length to further study the impact of fermentation conditions on PHB formation by strain MH 96. The findings are illustrated in Fig. 4. Strains were cultivated for different durations (12 to 96 h) at 150 rpm and 37 °C, as illustrated in Fig. 4A. The buildup of growth-associated PHB in strain MH 96 was seen during the exponential development phase (Fig. 4A). The highest PH PHB A production of 0.887 g/L and maximum DCW of 2.95 g/L were achieved at 96 h, with a recovery yield of 30.07% (w/w). Consequently, an incubation period of 96 h was selected as the optimal duration for subsequent investigations.

As indicated in Fig. 4B, a correlation was observed between increasing DCW and PHB generation as inoculum size concentration increased for strain MH 96. The highest DCW of 3.2 g/L and PHB production of 1.14 g/L were recorded at 96 h, with a maximum recovery yield of 35.62% (w/w). PHB accumulation and recovery yield decreased slightly at inoculum concentrations above and below this threshold, with the optimal yield achieved at 4% inoculum size.

Figure 4C illustrates that DCW remained relatively stable across a broad pH range (6.0 to 10.0), varying from 3.04 to 3.40 g/L after 96 h. At an initial pH of 5, DCW dropped to 2.43 g/L, indicating a significant decline. However, PHB production and recovery yield increased significantly with a rise in initial pH, peaking at pH 7, where 1.25 g/L and 36.76% (w/w) were recorded.

Figure 4D illustrates that strain MH 96 yielded the highest dry cell weight (DCW) of 3.34 g/L and polyhydroxyalkanoates (PHB) of 1.28 g/L at a sodium chloride concentration of 12.5% after 96 h of post-inoculation. This scenario resulted in a maximal recovery of 38.32% (w/w). Additionally, PHB accumulation and recovery yield exhibited a modest decline at sodium chloride concentrations both below and beyond this ideal threshold.

Figure 4E illustrates that strain MH 46 attained a dry cell weight (DCW) of 3.34 g/L after 96 h at 37 °C, yielding the highest PHB recovery rate of 38.32% (w/w) and a PHB production of 1.28 g/L. This result is consistent with the findings of Desouky, Abdel-Rahman [20] and Abdel-Rahman, Desouky [39] which reported that all optimized agents were prepared at 37 °C. Consequently, the ideal incubation temperature for maximizing PHA generation efficiency was determined. Ultimately, as illustrated in Fig. 4F, following 96 h at an agitation rate of 150 rpm, the maximum dry cell weight (DCW) of 3.34 g/L and polyhydroxyalkanoate (PHA) concentration of 1.28 g/L were achieved. This result is consistent with the findings of Desouky, Abdel-Rahman [20], which highlight the importance of stirring for effective mixing and mass and heat transfer. The effect of varying agitation rates (0.0 [fixed], 50, 150, 200, and 250 rpm) on the growth and PHA production of strain AZU-A2 was investigated. The highest dry cell weight (DCW) of 4.39 g/L and the maximum PHA concentration of 3.0 g/L were observed after 24 h at an agitation rate of 200 rpm.

### Results of response surface optimization utilizing PBD and BBD

Conventional optimization techniques are frequently laborious, susceptible to inaccuracies, and fail to enable the concurrent examination of several interacting variables [43]; nevertheless, data-driven treatment methodologies can be developed through statistical design. The product yield can be enhanced by optimizing the fermentation process. Multiple applications have been submitted for the statistical optimization of biological processes utilizing PBD and RSM [44]. Reports indicate that PHA yield optimization can be achieved by the RSM optimization technique [45] &[43]. This study employed PBD and BBD in multifactorial interaction experiments to determine the optimal fermentation conditions.

#### Results of PBD experiments

Fifty trials were conducted to evaluate eleven media components for their effects on PHB production. Trial eleven yielded the highest PHB, while trial four yielded the least (Table 5). Statistical analysis showed that inoculum size, (NH4) kHPO4 concentration, and initial pH were the most influential factors. ANOVA results for the Plackett–Burman design indicated a first-order model's determinant coefficient (R^2^) of 0.9998, with a significant F-value of 773.2, suggesting a 0.13% chance that this result is due to noise (Table 6). Additionally, a curvature F-value of 1131.76 pointed to substantial curvature in the design space, with only a 0.09% likelihood of being attributed to noise. Stepwise regression analysis was conducted using Design Expert 7.0, leading to the formulation of the following predictive equation for PHB yield (Y):

3 $$\text{Y}=1.43 - 0.1484\text{A }+1.667\text{E} - 004\text{B} - 0.39\text{C} - 0.77\text{D} + 0.25\text{F} - 0.30\text{G} - 0.27\text{H}+ 0.52\text{J} + 0.22\text{K} - 0.25\text{L} - 1.02\text{AB}$$

**Table 5:** Summarizes the Plackett–Burman experimental design utilized for screening the culture conditions affecting PHB production

Run	A	B	C	D	E	F	G	H	J	K	L	PHB (g/L)
1	−1	1	−1	1	1	−1	1	1	1	−1	−1	1.85
2	−1	1	1	−1	1	1	1	−1	−1	−1	1	2.1
3	1	−1	−1	−1	1	−1	1	1	−1	1	1	2.2
4	1	1	1	−1	−1	−1	1	−1	1	1	−1	1.47
5	−1	−1	1	−1	1	1	−1	1	1	1	−1	2.1
6	−1	−1	−1	1	−1	1	1	−1	1	1	1	0.78
7	0	0	0	0	0	0	0	0	0	0	0	1.92
8	1	−1	1	1	1	−1	−1	−1	1	−1	1	1.61
9	−1	−1	−1	−1	−1	−1	−1	−1	−1	−1	−1	1.47
10	0	0	0	0	0	0	0	0	0	0	0	1.92
11	1	1	−1	1	1	1	−1	−1	−1	1	−1	0.76
12	0	0	0	0	0	0	0	0	0	0	0	1.96
13	−1	1	1	1	−1	−1	−1	1	−1	1	1	0.55
14	1	−1	1	1	−1	1	1	1	−1	−1	−1	0.43
15	1	1	−1	−1	−1	1	−1	1	1	−1	1	1.86

A, Orange peel concentration (g/L); B, Temperature (^o^C); C, Sodium chloride (%); D, Incubation time (hr.); E, Inoculum size (%) (v/v); F, Agitation rate (rpm); G, MgCl_2_.6H_2_O (g/L); H, MgSO_4_.7H_2_O (g/L); J, (NH_4_)_2_HPO_4_ (g/L); K, Methanol (co-substrate) (g/L); L, Initial pH

**Table 6:** provides the results of the main effect analysis for each factor in the Plackett–Burman experimental design

Source	Sum of Squares	Df	Mean Square	F Value	P Value
Model	4.535	11	0.412	773.019	0.0013
Orange peel concentration	0.023	1	0.023	42.576	0.0227
Temperature	0.000	1	0.000	0.001	0.9823
Sodium chloride	0.930	1	0.930	1743.451	0.0006
Incubation time	3.588	1	3.588	6728.000	0.0001
Agitation rate	0.360	1	0.360	675.281	0.0015
MgCl_2_.6H_2_O	0.534	1	0.534	1001.281	0.0010
MgSO_4_.7H_2_O	0.443	1	0.443	830.281	0.0012
(NH_4_)_2_HPO_4_	1.614	1	1.614	3026.420	0.0003
Methane	0.282	1	0.282	528.125	0.0019
PH	0.386	1	0.386	723.901	0.0014
AB	1.375	1	1.375	2578.101	0.0004
Curvature	0.6036	1	0.6036	1131.7601	0.0009
Pure Error	0.0011	2	0.0005		
Cor Total	5.1397	14			
Detemination cofficient R^2^ = 0.9998; Adjusted determination R^2^ = 0.9985; Coefficient of variation CV = 1.5%					

#### Response surface methodology

According to the results above, PHB production was greatly impacted by three important factors: inoculum size, (NH_4_)_2_HPO_4_, and initial pH. These factors were further analyzed using Box-Behnken Design (BBD), as detailed in (Table 7). The resulting second-order polynomial equation is expressed as:

4 $$\text{Y}=+2.23 - 0.011\text{A}+ 0.030\text{B }+ 0.087\text{ C }+ 0.32\text{AB }- 0.35\text{AC }+ 0.26\text{BC}+{1.82\text{A}}^{2} + {0.97\text{B}}^{2} + {0.98\text{C}}^{2}$$

where A, B, and C represent the concentrations of inoculum size, (NH_4_)_2_HPO_4_, and initial pH, respectively, while Y corresponds to PHB production (g/L). The coefficients indicate the influence of linear, interactive, and quadratic terms on PHB yield. ANOVA analysis alongside F-tests was employed to evaluate the statistical significance of the model (Table 8).

**Table 7:** Experimental design and results of box–behnken optimization experiment

Trials	A (inoculum size)		B (NH_4_)_2_HPO_4_		C (Initial pH)		PHB (g/L)
	**Coded level**	**Real level (%)**	**Coded level**	**Observed**	**Coded level**	**Real level (** ^**o**^ **c)**	**Observed**
**1**	1	6	0	1.5	−1	6.5	5.25
**2**	−1	2	−1	2	0	7	5.41
**3**	0	4	1	2	1	7.5	4.48
**4**	−1	2	0	1.5	−1	6.5	4.45
**5**	1	6	1	2	0	7	5.284
**6**	0	4	1	2	−1	6.5	3.97
**7**	−1	2	0	1.5	1	7.5	5.369
**8**	0	4	−1	2	1	7.5	3.87
**9**	0	4	−1	2	−1	6.5	4.4
**10**	−1	2	1	2	0	7	4.805
**11**	0	4	0	1.5	0	7	4.32
**12**	0	4	0	1.5	0	7	4.35
**13**	0	4	0	1.5	0	7	5.02
**14**	0	4	0	1.5	0	7	4
**15**	1	6	0	1.5	1	7.5	4.92
**16**	1	6	0	2	−1	7	4.62
**17**	−1	4	−1	1.5	0	7	5.72

A, inoculum size (%); B, (NH_4_)_2_HPO_4_ (g/L); and C, Initial pH

**Table 8:** ANOVA results for response surface

Source	Sum of Squares	Df	Mean Square	F Value	*P*- Value
Model	25.497	9	2.833	187.927	< 0.0001
A- inoculum size	0.001	1	0.001	0.069	0.8008
B-(NH_4_)_2_HPO_4_	0.007	1	0.007	0.474	0.5135
C-PH	0.061	1	0.061	4.063	0.0837
AB	0.403	1	0.403	26.706	0.0013
AC	0.476	1	0.476	31.582	0.0008
BC	0.270	1	0.270	17.937	0.0039
A^2^	14.022	1	14.022	930.131	< 0.0001
B^2^	4.002	1	4.002	265.446	< 0.0001
C^2^	4.004	1	4.004	265.582	< 0.0001
Residual	0.106	7	0.015		
Lack of Fit	0.106	3	0.035		
Pure Error	0.000	4	0.000		
Cor Total	25.603	16			

Determination coefficient R^2^ = 0.9959; Adjusted determination R^2^ = 0.9906; Coefficient of variation CV = 3.06%; model are significant

**Fig. 5:**
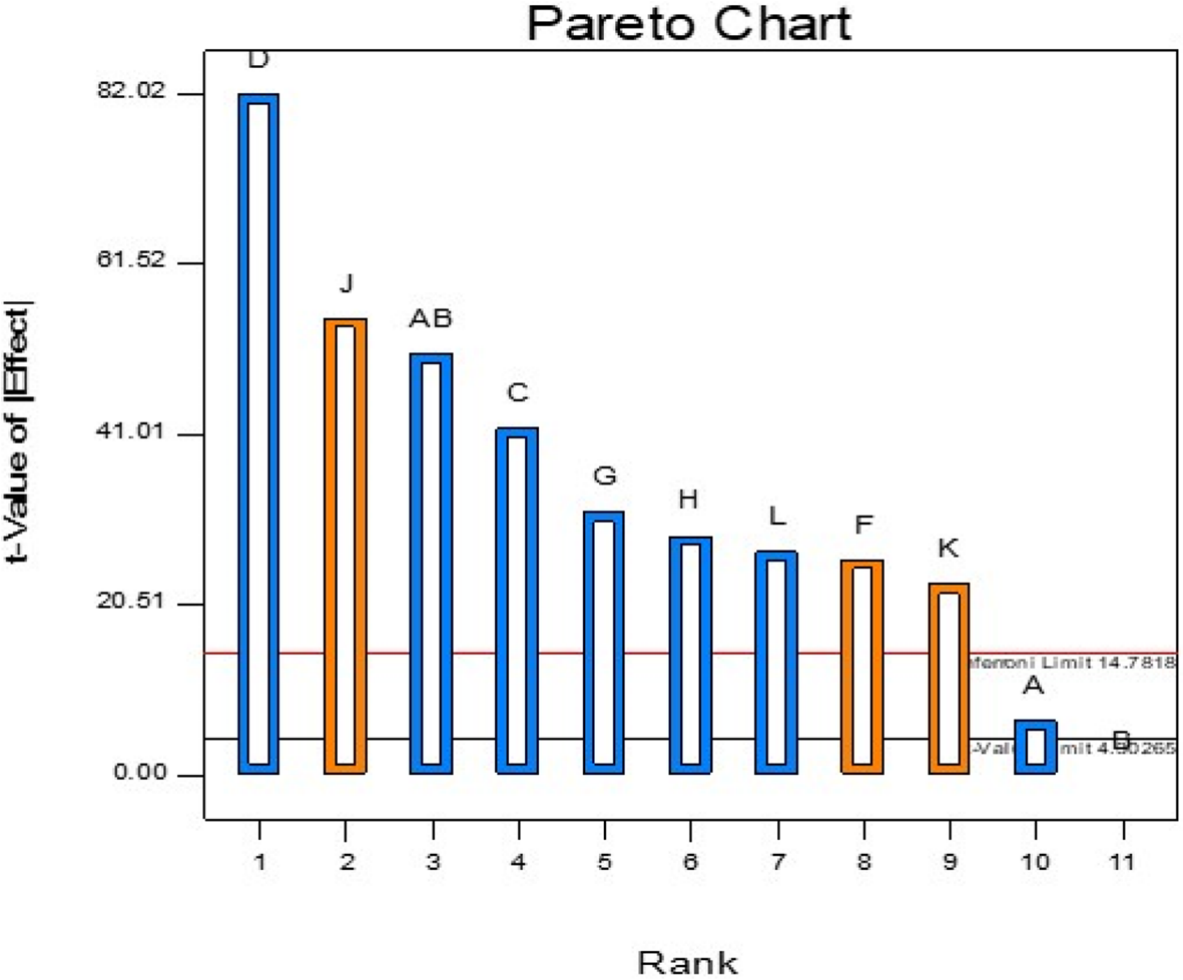
The Pareto chart shows significant factors affecting PHB production by strain MH 96

The model demonstrated a high degree of reliability, with an R^2^ value of 0.9959, suggesting that it accounts for 99% of the variability in the response (Fig. 5). The significance of the model is supported by an F-value of 187.93, with only a 0.01% likelihood that such an F-value could arise from noise. Model terms with"Prob > F"values below 0.0500, including AB, AC, BC, A^2^, B^2^, and C^2^, were identified as significant.

**Fig. 6:**
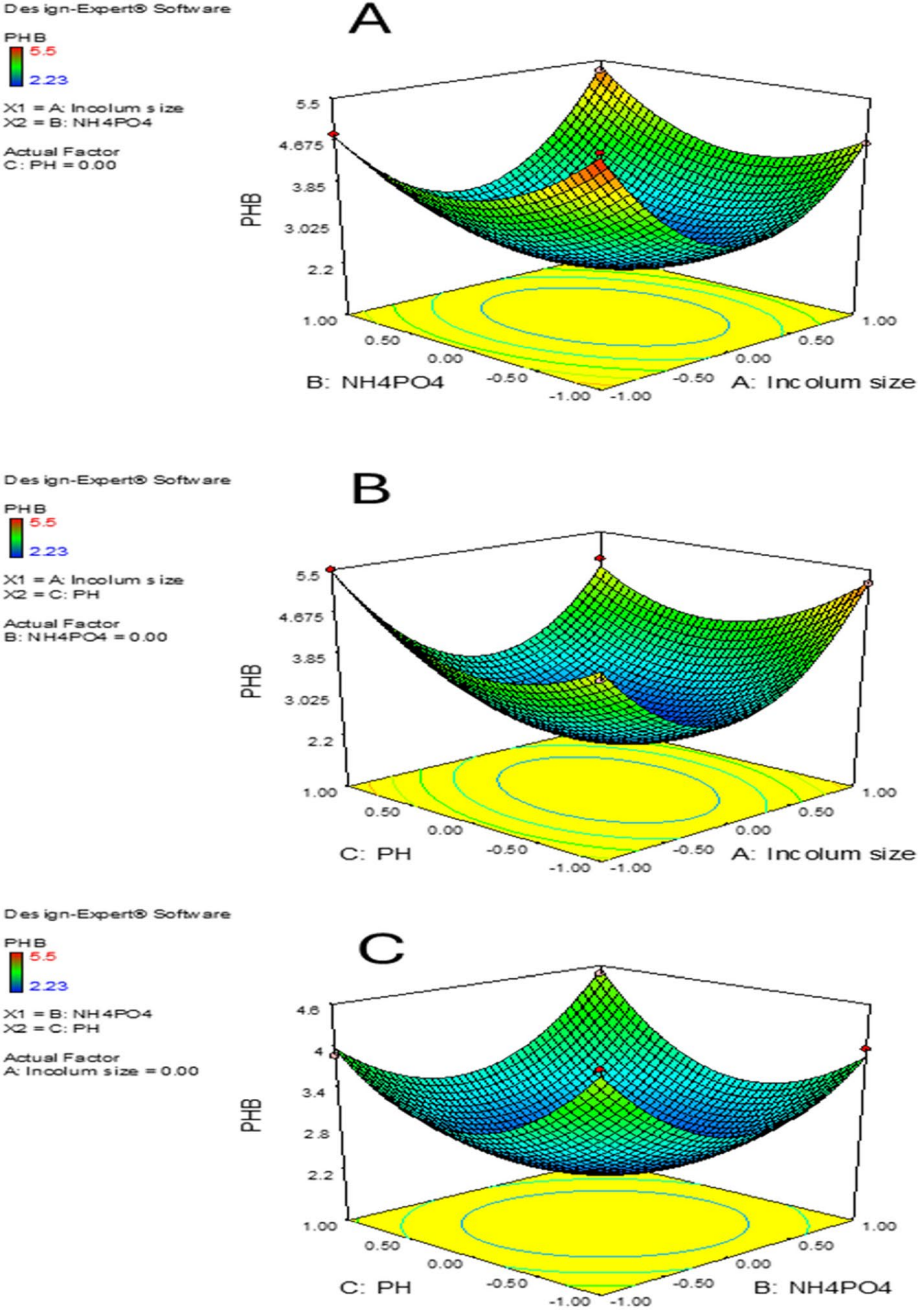
Response surface plots illustrating the effects of three variables on PHB production. (A) Interaction between (NH₄)₂HPO₄ concentration and inoculum size, (B) interaction between pH and inoculum size, and (C) interaction between pH and (NH₄)₂HPO₄ concentration

For strain, MH 96, which utilizes orange peel as a carbon source for PHB synthesis, the optimal parameters identified through Design Expert 7.0 software were the final revision is inoculum size 1.74, (NH_4_)_2_HPO_4_ concentration 1.0 and pH 6.37 (Fig. 6).

#### Experimental verification based on the optimization results

The response surface analysis identified the optimal conditions for PHB production: an inoculum size of 1.74, an (NH₄)₂HPO₄ concentration of 1.0 g/L, and a pH of 6.37, resulting in a maximum PHB yield of 5.94 g/L. To validate these conditions, fermentation trials were conducted three times under the optimized parameters, followed by bacterial harvesting after 72 h to measure PHB production. The close agreement between experimental and predicted results confirms the reliability of the quadratic model in assessing the combined effects of multiple factors on PHB biosynthesis. The 3D response surface plot displayed a convex shape, emphasizing the interplay between inoculum size, pH, and (NH₄)₂HPO₄ concentration in optimizing PHB yield (Fig. 3). The observed trends suggest that these parameters significantly influence microbial metabolism, nutrient assimilation, and polymer accumulation. The strong correlation between the model's predictions and actual results highlights the effectiveness of the RSM model in optimizing PHB production. These findings reinforce the model’s predictive accuracy and provide a basis for scaling up PHB biosynthesis using cost-efficient and renewable feedstocks.

### Characterization of PHB

#### GC–MS analysis

**Fig. 7:**
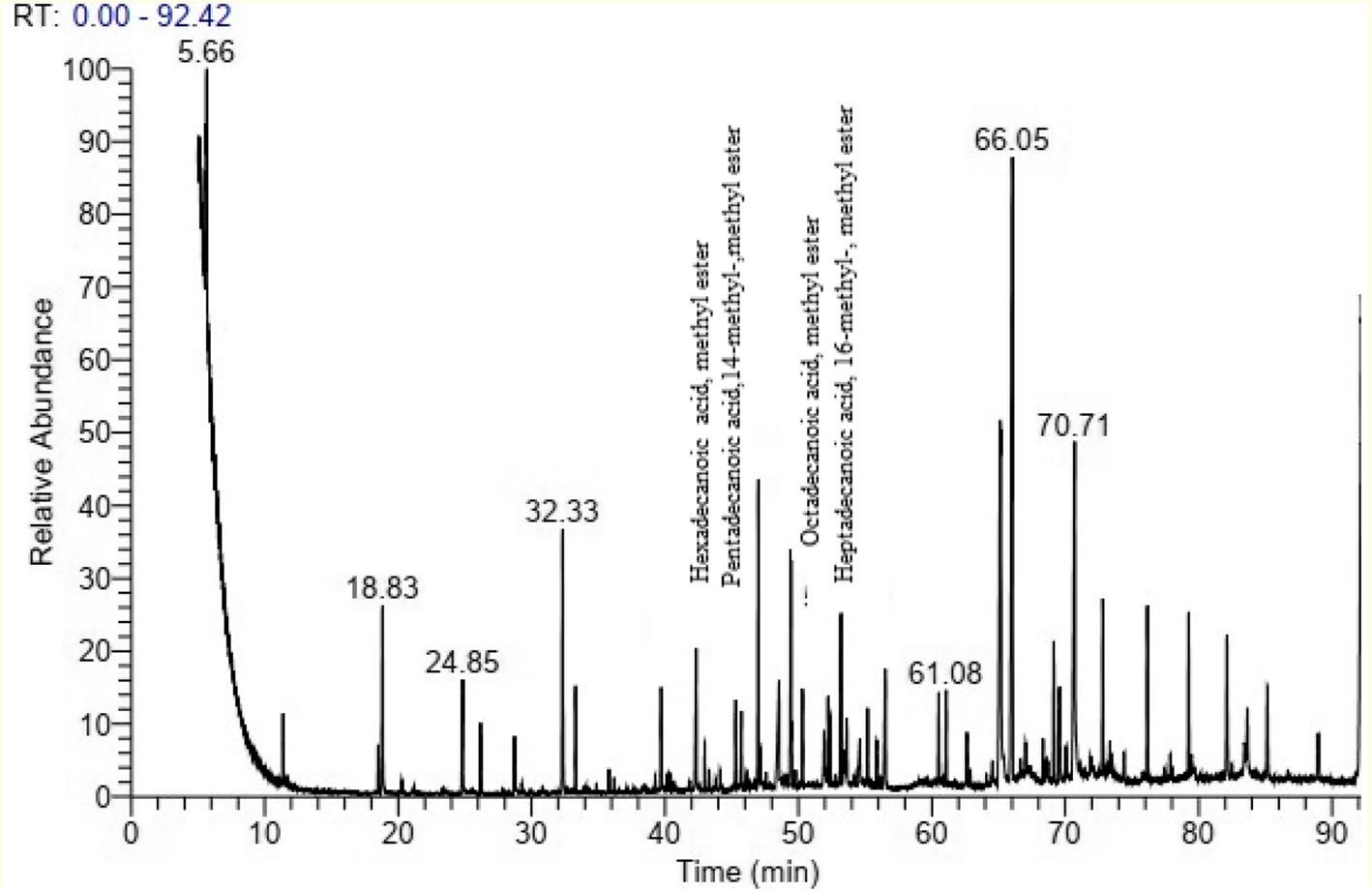
GC–MS spectral chromatogram of derivatives PHB obtained from *H. meridiana*

The molecular structure of PHB can be effectively measured and described by gas chromatography. For GC analysis, PHB must be depolymerized into acids, diols, or esters [46]. Following methanolysis of the PHB sample, the methyl esters exhibited fragmentation patterns in GCMS, facilitating the identification of the resultant PHB derivatives. Four principal peaks were detected in the biopolymer extract of *H. meridiana* (Fig. 7), with retention periods of 46.76 min, 46.76 min, 52.94 min, and 52.94 min, respectively. Dimers of ç-hydroxybutyric acid, crotyl ester, and ç-hydroxybutyrate, along with 2-butenoic acid, 1-methyl ethyl ester, tetradecane, hexadecanoic acid, methyl ester, and docosanoic acid, 8,9,13-trihydroxy methyl ester, are included. Table 9 illustrates the primary peaks, confirming the existence of polyhydroxybutyric acid (PHB) in *H. meridiana* samples. This finding corresponded with Mandragutti, Jarso [7] and Hong, Song [47].

**Table 9:** List of PHB derivatives detected in GC–MS

Compounds structure	Molecular formula	MW (g/mol)	Probability	Peak area%	Compounds name	RT	Code
	C_17_H_34_O_2_	220	16.61	0.30	Hexadecanoic acid, methyl ester	46.76	**A**
	C_17_H_34_O_2_	270	30.44	0.30	Pentadecanoic acid,14-methyl-,methyl ester	46.76	**B**
	C_19_H_38_O_2_	298	14.32	0.45	Octadecanoic acid, methyl ester	52.94	**C**
	C_19_H_38_O_2_	198	31.19	0.45	Heptadecanoic acid, 16-methyl-, methyl ester	52.94	**D**

#### Fourier transform-infrared spectroscopy (FTIR)

**Fig. 8:**
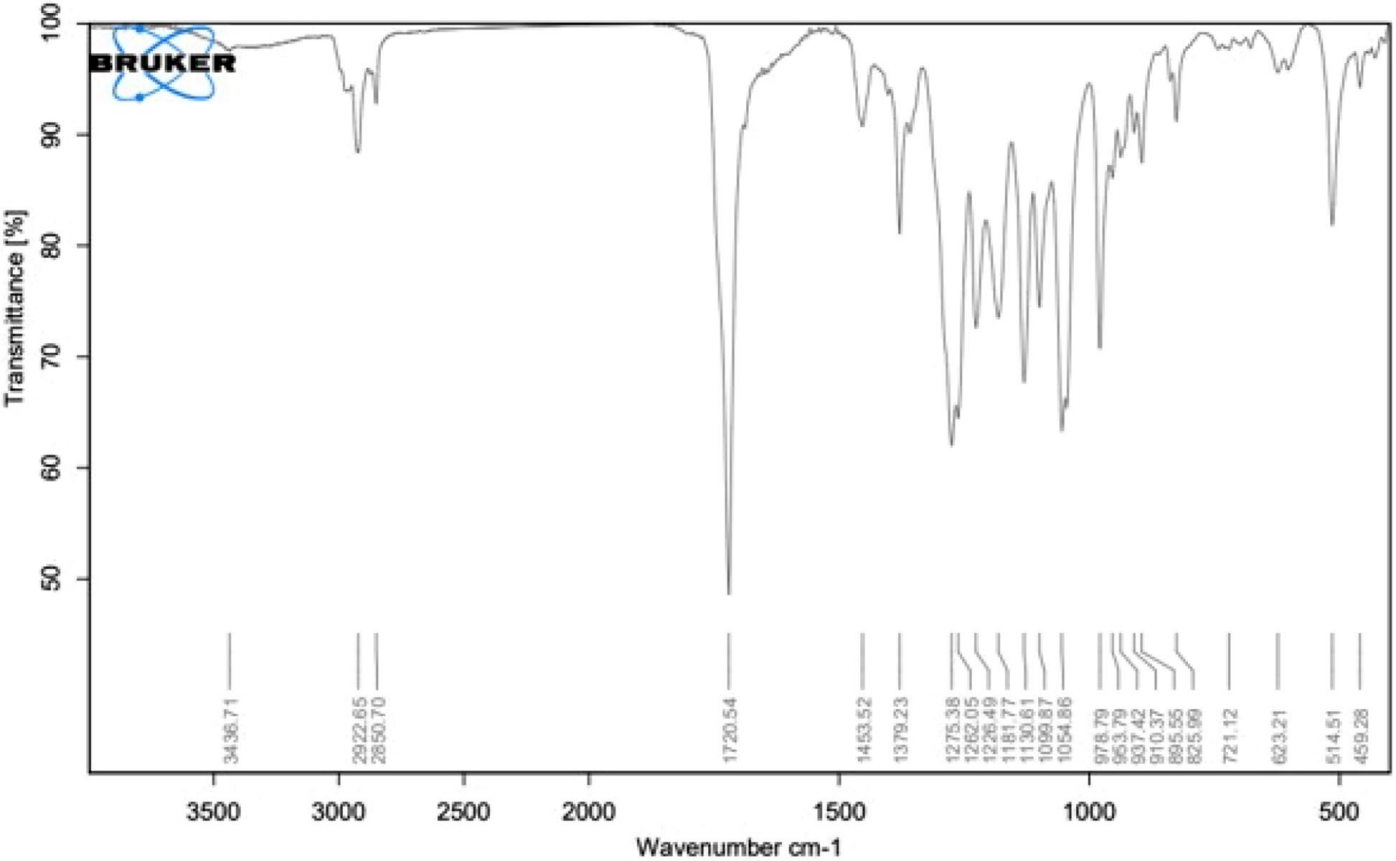
Fourier transform infrared spectra analysis of PHB produced by *H. meridiana*

The IR spectrum of the PHB compound (KBr, υmax in cm⁻^1^) displayed characteristic absorption bands at 3436.71 cm⁻^1^ corresponding to O–H stretching, 2922.65 cm⁻^1^ for C–H stretching, and a strong band at 1720.54 cm⁻^1^ indicative of C = O stretching. Additional bands at 1453.52 and 1275.38 cm⁻^1^ were attributed to methylene bending, as shown in Fig. 8. These results agree with [20] showed absorption bands at 3453 cm^−1^ (OH stretching), 2980 cm^−1^ (C-H stretching), 1448 and 1285 cm^−1^ (methylene bending), and 1736 cm^−1^ (C = O stretching). Furthermore, the outcomes concur with the latest research by [48], It had the C = O characteristic band, which is the most significant one for PHB isolated from *Haloferax mediterranei*. We noticed this band at 1720–1740 cm^−1^. This result aligns with the findings of [49], which present the spectra of three commercial PHB standards. A distinct peak area, corresponding to PHB concentration, is observed within the region of 1728 cm⁻^1^ to 1744 cm⁻^1^. Moreover, the results align with Furthermore, the results above correspond with [7] in which *B. paraconglomeratum*, the carbonyl group was centered at 3019 cm^−1^, which indicates the presence of longer aliphatic chains. The stretches in 1709 and 1362 cm^−1^ indicate the C = O, C-H bending or CH_3_.The obtained IR spectra showed typical ester C-O bonds at 1217 cm.^−1^.[35]

#### Nuclear magnetic resonance (NMR)

**Fig. 9:**
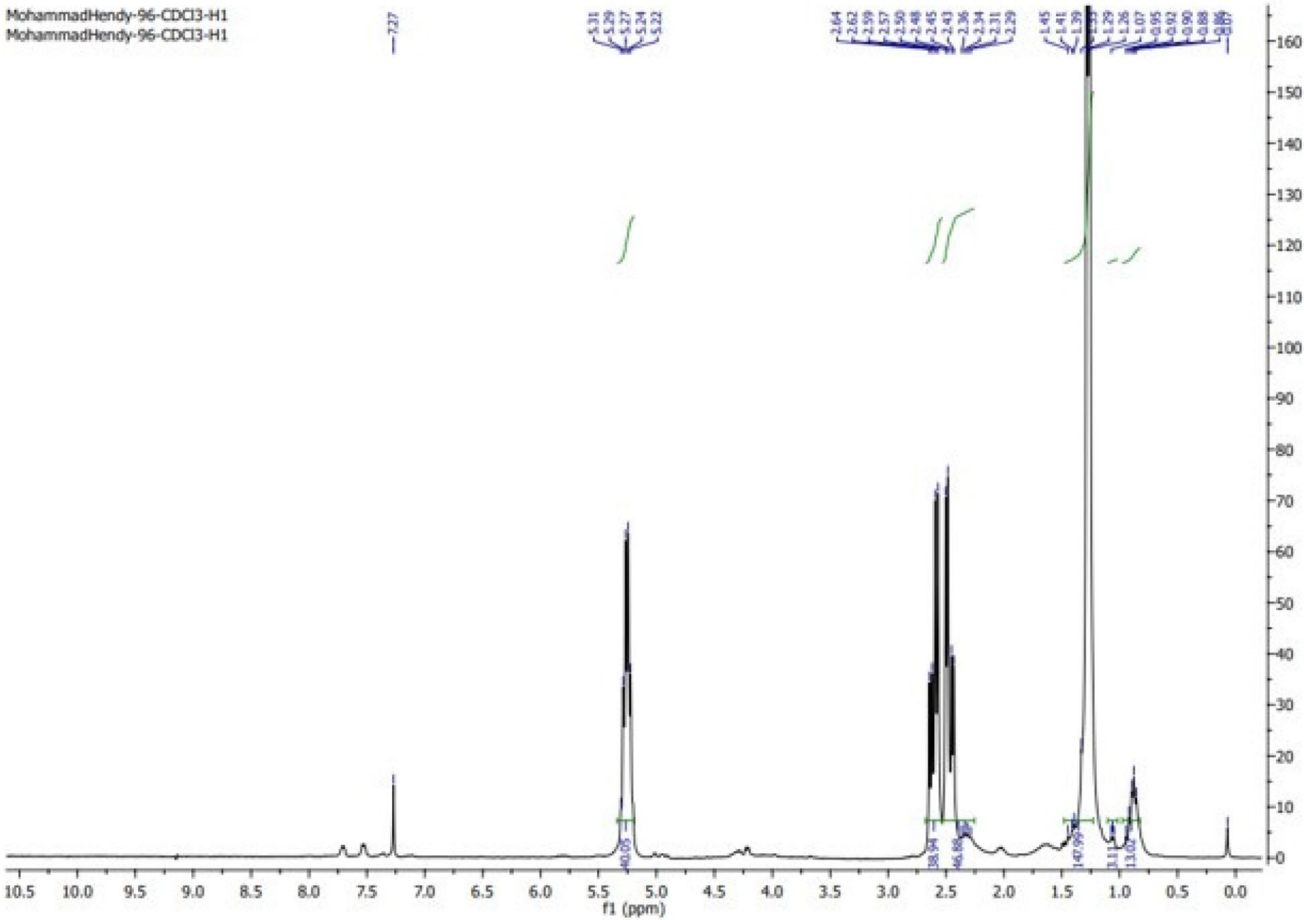
^1^H-NMR spectra of PHB produced by *H. meridiana*

**Fig. 10:**
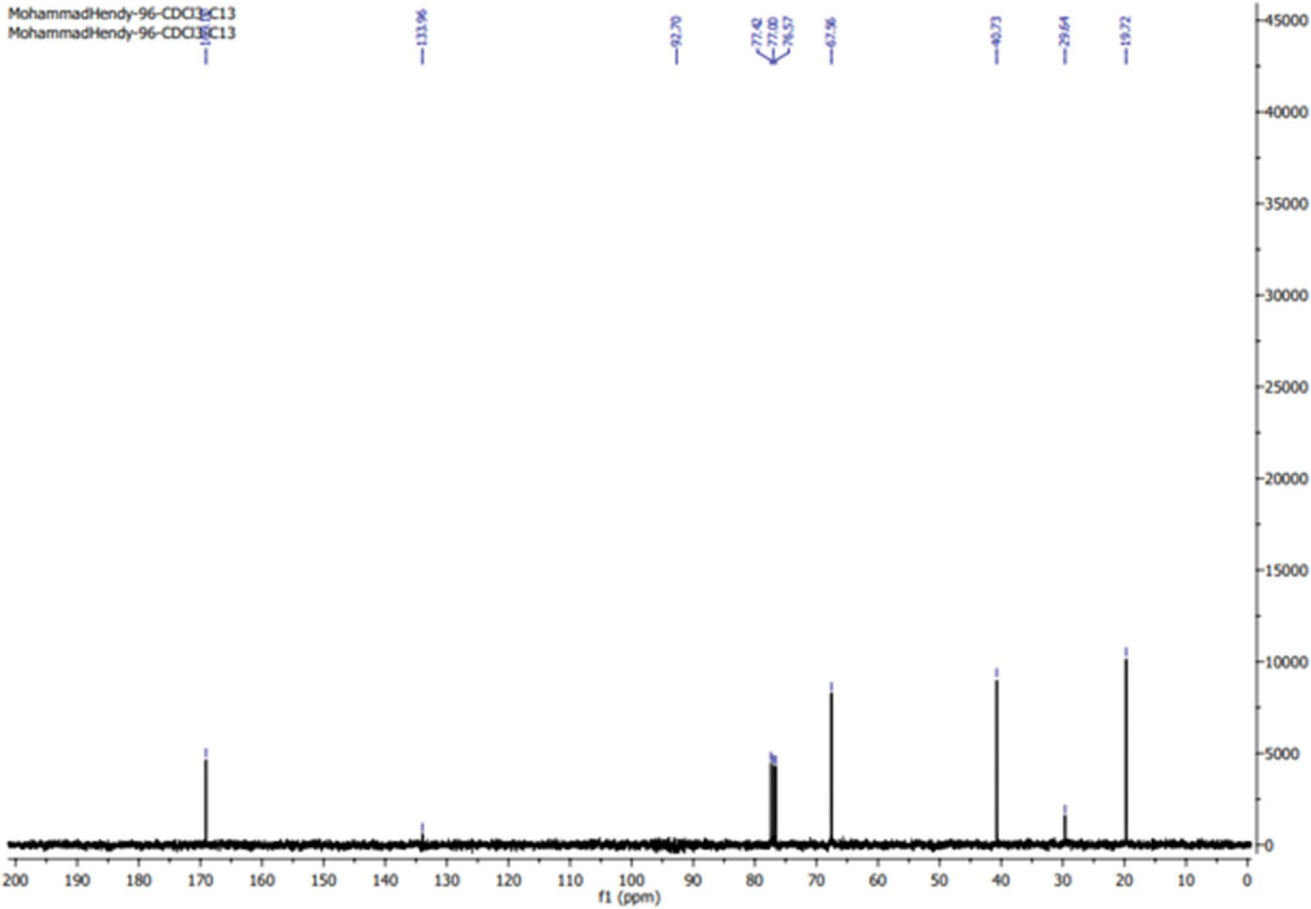
^13^C-NMR spectra of PHB produced by *H. meridian*

The ^1^H-NMR spectrum of the PHB isolated from strain MH 96 (Fig. 9) displayed characteristic signals corresponding to the structure of PHB s. A doublet at 1.33 ppm was assigned to the methyl (–CH₃) group, coupled to a single proton, while a doublet of quadruplets at 2.44 ppm was attributed to the methylene (–CH₂) group adjacent to an asymmetric carbon. Additionally, a signal at 5.21 ppm corresponded to the methine (–CH) group. Since these signals have been previously found in the PHB standards [50], they are similar to our sample, it can be confirmed that the biopolymer was synthesized by *H. meridiana* is a PHB. The measured chemical shifts and assignments aligned closely with a genuine PHB sample from Aldrich, validating the extracted biopolymer as poly-3-hydroxybutyric acid, further corroborated by the ^13^C NMR spectrum. Additionally, the ^13^C NMR spectra (Fig. 10) displayed peaks at 19.72, 40.73, 67.55, and 169.116 ppm, which correspond to the resonances for (–CH₃), (–CH₂–), (–CH–), and (–C–), respectively. The synthesized polymer was verified as PHB using the resonances of the methyl, methylene, methine, ester groups, and carbonyl carbon atoms. This data corroborates with [7] the ^1^H NMR spectra of *B. paraconglomeratum* PHB extracts displayed resonances for the hydroxybutyrate side groups: a peak for (-CH3) at 1.224 ppm, a singlet for (-CH-) at 5.163 ppm, and doublets for (CH2) at 2.164 and 2.072 ppm. The 13 C NMR spectra (Fig. 10) exhibited peaks at 19.71, 40.75, 67.63, and 169.43 ppm, corresponding to (CH3), (–CH2–), (–CH–), and (–C–), respectively. The resonances of methyl, methylene, methane, ester groups, and the carbonyl carbon atom validated that the produced polymer was PHB.

#### X-ray diffraction

**Fig. 11:**
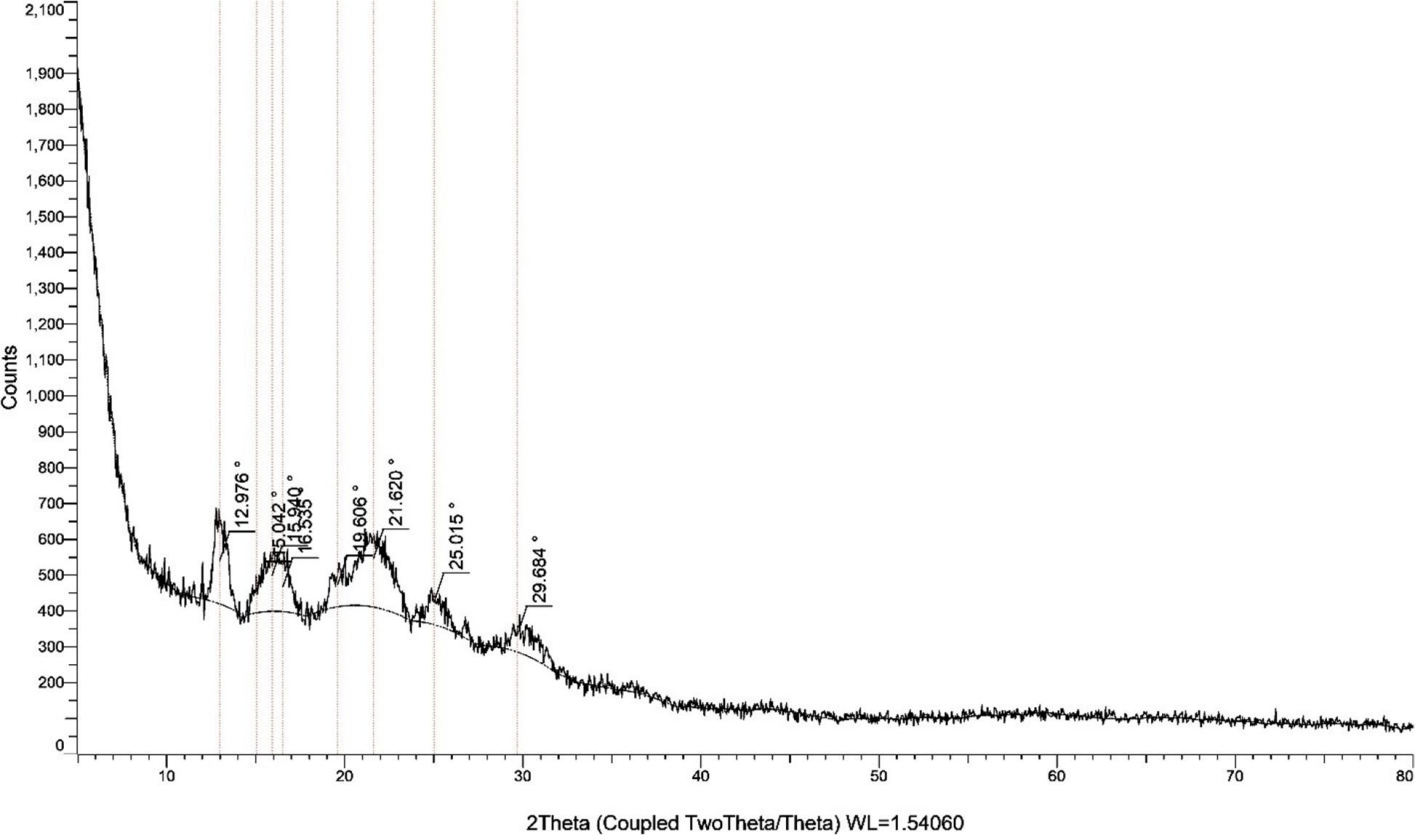
XRD graph represents the crystalline characteristic of PHB for the isolate *H. meridiana*

X-ray diffraction (XRD) analysis further confirmed the crystalline nature of the polymer. The XRD profile showed well-defined diffraction peaks at 2θ values of 12.976°, 15.042°, 15.940°, 16.535°, 19.606°, 21.620°, 25.015°, and 29.684°, which align with the typical crystalline pattern of PHB (Fig. 11). These results collectively affirm the successful biosynthesis and isolation of PHB by *H. meridiana*. The results correlate with those reported by [43], which detected analogous peaks in the XRD data for strain L17, corresponding to the (020), (110), (101), (121), and (002) planes, with peak positions at 13.6°, 17.24°, 21.4°, 25.7°, and 30.5°. The XRD pattern of the polymers derived from orange peel closely corresponded with the standard PHB, exhibiting characteristic crystalline peaks of the polymer.

#### Thermal characterization of the purified polymer

**Fig. 12:**
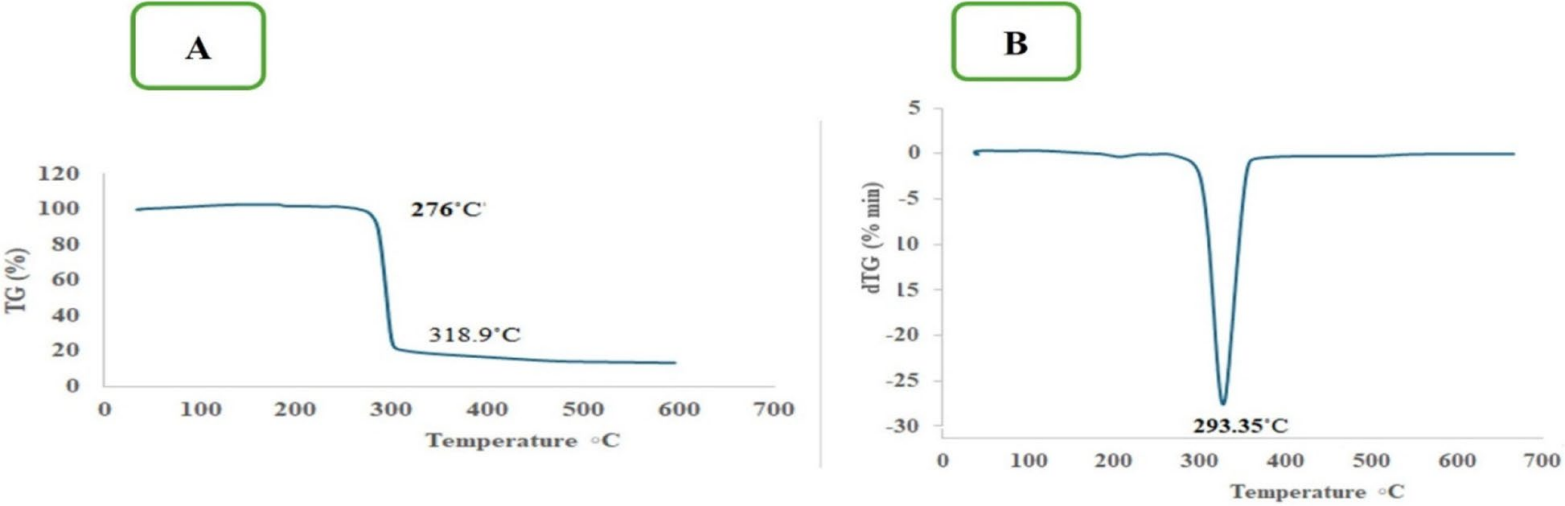
Thermal analysis of *H. meridiana* PHB by TGA

TGA analyses are essential for establishing the processing thresholds of thermoplastic substances, especially for PHB, which possesses a limited processing range [51]. TGA studies are essential for understanding the processing limits of thermoplastic materials like PHB, which has a narrow processing window. The TGA analysis revealed a two-step decomposition process: an initial weight loss between 30 and 100 °C due to water loss, followed by a second phase starting at 276 °C, with complete degradation at 318.9 °C. At this temperature, ester bonds break, producing shorter chain fragments with carboxylic acid and olefinic terminal groups. The decomposition temperature (Td) for PHB from *H. meridiana* was found to be 293.35 °C, supported by the DTG curve showing a peak weight loss rate at this temperature, indicating good quality PHB.At this temperature, ester bonds break, producing shorter chain fragments with carboxylic acid and olefinic terminal groups. The decomposition temperature (Td) for PHB from *H. meridiana* was found to be 293.35 °C, supported by the DTG curve showing a peak weight loss rate at this temperature, indicating good quality PHB. (Fig. 12A). At this temperature, the ester bonds were broken, resulting in shorter chain fragments containing carboxylic acid and olefinic terminal groups. [52]. The decomposition temperature (Td) for PHB produced by *H. meridiana* was determined to be 293.35 °C. This finding is supported by the DTG curve, which indicated a peak weight loss rate at this temperature, suggesting that the PHB produced is of good quality (Fig. 12B). Moreover, these results align closely with reference samples from prior research (Huanget al., 2023).

**Fig. 13:**
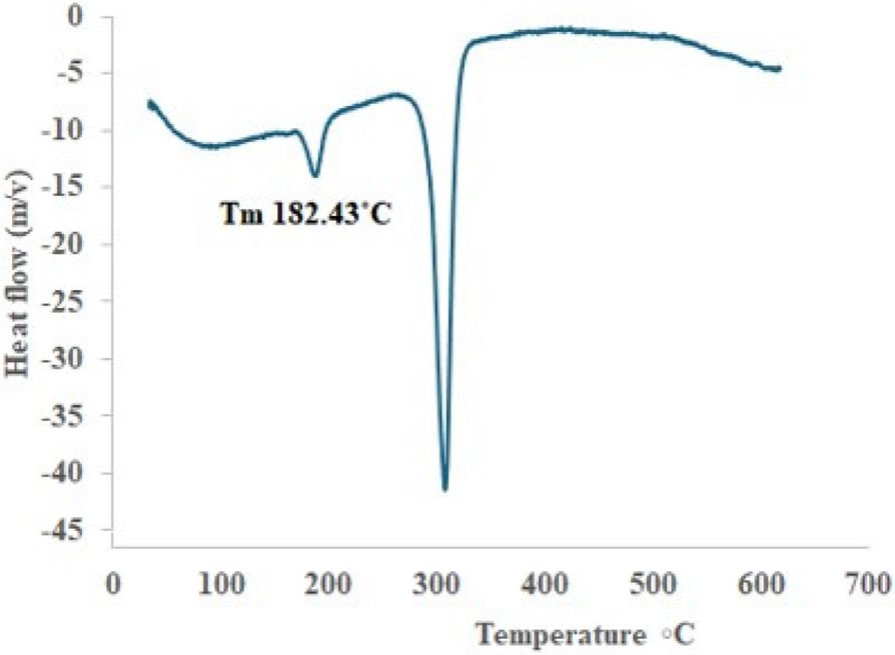
DSC analysis of PHB produced by *H. meridiana*

Subsequent DSC analysis (Fig. 13) indicated that the isolated PHB displayed a melting temperature (Tm) of 182.43 °C. The melting temperature is analogous to PHB derived from alternative sources, including EPPJ and glucose, which exhibited Tm values of 172 °C and 175 °C, respectively. These findings align with traditional PHB, which has a documented Tm of 176 °C [35]. The higher thermal stability of polyhydroxyalkanoates (PHAs) is a crucial aspect of their polymerization processes, signifying the polymer's capacity to endure high temperatures [35].

## Research needs and perspectives

Polyhydroxybutyrates (PHBs) are biodegradable, eco-friendly polymers with applications in medicine, packaging, nanotechnology, and agriculture. Despite their potential, commercialization faces challenges due to inefficient production, high costs, and complex extraction processes. The reliance on expensive raw materials and energy-intensive fermentation further limits scalability [54]. Enhancing microbial strains, optimizing fermentation, and improving downstream processing are crucial for making PHBs more cost-effective. While ongoing research aims to reduce production costs and environmental impact, significant hurdles remain for large-scale adoption as a viable alternative to conventional plastics [53]. [54].

## Conclusion

This research revealed a promising candidate strain, *H. meridiana*, capable of producing PHB from orange peel. The synthesized PHB has been characterized as polyester using GC–MS, Fourier transform infrared (FTIR) spectroscopy, NMR spectroscopy, X-ray diffraction, and thermal analysis (DSC and TGA). The considerable production of PHB indicates that orange peel is a significant waste byproduct for research and development in bioplastics. After doing single-factor optimization and response surface optimization, the yield of PHB rose to 5.94 g/L.

## Data Availability

Data availability statement: the datasets analyzed during the current study are available in the NCBI GenBank database repository with the accession number of PP826284, https://www.ncbi.nlm.nih.gov/nuccore/PP826284.
